# Nanopore current transduction analysis of protein binding to non-terminal and terminal DNA regions: analysis of transcription factor binding, retroviral DNA terminus dynamics, and retroviral integrase-DNA binding

**DOI:** 10.1186/1471-2105-8-S7-S10

**Published:** 2007-11-01

**Authors:** Stephen Winters-Hilt, Amanda Davis, Iftekhar Amin, Eric Morales

**Affiliations:** 1 Department of Computer Science, University of New Orleans, New Orleans, LA 70148, USA; 2 Research Institute for Children, Children's Hospital, New Orleans, LA 70118, USA

## Abstract

**Background:**

Synthetic transcription factors (STFs) promise to offer a powerful new therapeutic against Cancer, AIDS, and genetic disease. Currently, 10% of drugs are of this type, including salicylate and tamoxifen. STFs that can appropriately target (and release) their transcription factor binding sites on native genomic DNA provide a means to directly influence cellular mRNA production. An effective mechanism for screening amongst transcription factor (TF) candidates would itself be highly valued, and such may be possible with nanopore cheminformatics methods.

**Results:**

It is hypothesized that binding targets on channel-captured molecules, that are well away from the channel-captured region, can be monitored insofar as their binding status, or history, is concerned. The *first *set of experiments we perform to explore this "transduction" hypothesis involve *non-terminal *dsDNA binding to protein (DNA TATA box receptor binding to TBP), where we show new experimental results and application of a new cheminformatics data analysis method. In the *second *series of experiments to explore the transduction hypothesis we examine *terminal *(blunt-ended) dsDNA binding to protein. We show experimental results before and after introduction of HIV's DNA integrase to a solution of bifunctional "Y" shaped aptamers that have an HIV consensus terminus exposed for interaction.

**Conclusion:**

X-ray crystallographic studies have guided our understanding of DNA structure for almost a century. It is still difficult, however, to translate the sequence-directed curvature information obtained through these tools to actual systems found in solution. With a nanopore detector the sequence-dependent conformation kinetics of DNA, especially at the DNA terminus, can be studied in a new way while still in solution and on a single molecule basis.

## Introduction

Nanopore detector measurements consist of sensitive observations of ionic current flow through a single nanopore with blockading analytes present. In early nanopore detection work, the data analysis problems were also of a familiar "Coulter event" form (resistive pulse measurements, familiar from cell counting with the Coulter counter [1]). A more informative setting is possible, however, with nanometer scale channels due to non-negligible interaction between analyte and channel. In this situation the blockading molecule need not necessarily provide a *single*, fixed, current reduction in the channel. One possible result of multiple bound states for a channel-captured molecule, for example, is to modulate the ion flow through the channel by imprinting the molecule's binding interactions (with the channel) and conformational kinetics on the confined channel flow environment (appearing as multiple blockade levels). Single molecules of duplex DNA, for example, are too large to translocate through the alpha-Hemolysin channel, and enter only about nine of ten base-pairs into the detector's larger *cis *side vestibule, before reaching the internal limiting aperture beyond which they cannot translocate further. At the limiting aperture the electrophoretic field strength is concentrated, and it is in this high-strain environment, with binding possibilities to the adjoining amino acids near the limiting aperture, etc., that ion flow activity is most sensitively influenced. The end of the captured molecule can, thus, directly modulate the ionic current flowing through the critical limiting aperture. The ionic current modulation information can be distinctive to the blockading molecule, a "signature".

Very large biological pores (1–2 nm) can be used as the basis for a "nanopore detector" sensing device. This is a relatively new experimental approach that dates from the pioneering experiments of Bezrukov et al. [2,3]. Their work proved that detection could be reduced to the molecular scale and applied to polymers in solution. A seminal paper, by Kasianowicz et al., 1996 [4], then showed that *individual *DNA and RNA polymers could be detected via their translocation blockade of a nanoscale pore formed by α-Hemolysin toxin (where the DNA movement is electrophoretically driven by an applied potential). Machine Learning based pattern recognition methods have since been used to discriminate between the four Watson-Crick base-pairs termini at the ends of individual DNA hairpin molecules [5], as well as to measure DNA duplex stem length, base pair mismatches, and loop length [6].

Taken further, nanopore detection can be augmented to perform nanopore current transduction detection (see [7] in this journal for further details). The main element underlying the transduction augmentation is the introduction of a specially designed "multi-level blockader" molecule. With the transduction experiments described here we explore the nanopore channel's capability for detecting protein binding to exposed non-terminal and terminal regions of a channel-captured DNA molecule. The DNA molecules central to the nanopore detector experiments performed here are also referred to as a bifunctional aptamers due to their two special binding functionalities: (i) to be captured by the channel and elicit an "informative" signal modulation; and (ii) to have a non-captured portion that is free to bind to a specific target (TBP or HIV integrase in the work described here).

Two types of DNA molecules are examined: (i) DNA hairpins; and (ii) three-way DNA junctions – "Y-aptamers" (two sub-classes studied). The molecules were designed for simple nanopore-based examination (a single orientation of capture, when possible, for example) and enhanced pattern recognition with our channel current cheminformatics software.

The first DNA molecule examined with our Nanopore Detector (see Methods) is a bifunctional three-way DNA junction, or "Y-aptamer". Aptamers are nucleic acid species that have been engineered to bind to various molecular targets such as small molecules, proteins, and nucleic acids. Aptamers are advantageous for biotechnological applications, because they are readily produced by chemical synthesis and possess desirable storage properties. The bifunctionality of this DNA aptamer is accomplished in part by certain steric constraints arising from its "Y" shape. According to our design, the blunt-ended terminus corresponding to the base of the Y will be captured and carefully perched over an internal limiting aperture in the channel detector (see Methods). The two remaining termini, the "arms" of the Y-shaped aptamer, are capped with thymine loops, preventing them from entering the narrow channel. The benefit of this particular arrangement is that now the detector can be operated in a way sensitive to binding events on the extremities of the captured DNA molecule. Thus, one or both arms can then be outfitted with a binding site to fulfill the molecule's additional function, which would allow an instance of non-terminal dsDNA binding to be appraised. In our case, a TATA box binding receptor is placed approximately at the mid-point of one of the aptamer arms. The experiment is also repeated, with the receptor arm elongated several base pairs for more distal receptor placement from the channel environment, in order to ensure accommodation for the TATA binding protein (TBP), with similar indication of binding in our experiments (see Results). An investigation of this kind has relevance to protein-based dsDNA binding, such as for TBP and multi-component TFs in general, as well as to ssDNA-based binding to non-terminal dsDNA regions for both protein and DNA/RNA-based STFs.

The second DNA molecule studied is a DNA hairpin in the family of blunt-ended duplex DNA termini with the sequence GAXX-3'. DNA sequences terminating either GAXX-3' or CAXX-3' are of intense biological and medical interest since such termini are found at the end of the retroviral DNA of HIV. GAXX-3'is a less common variant, with which a prominent decrease in integration activity is observed [8]. Part of HIV's attack is mediated by the binding of its retroviral DNA terminus to integrase for insertion into the host genome (with the XX-regions above removed in the process). The HIV terminus and its variants are shown to have exceptional flexibility and channel-binding interactivity in preliminary nanopore detector analysis [9].

For the third DNA molecule studied we merge the study cases of the first and second molecules: we have placed the HIV consensus terminus, that was studied in the second DNA molecule, at the end of the Y-aptamer arm (that was first DNA molecule's TATA arm) – where it is exposed for binding to integrase (if in a properly oriented base-captured orientation of the Y-aptamer). This permits direct examination of protein binding to the terminal DNA region.

## Background

The Background that follows encompasses three subjects relevant to the Nanopore Detector examination of DNA-protein binding interactions: (1) Transcription Factors (TFs), their Binding Sites (TFBSs) in DNA, and possible drug mechanisms; (2) Nanopore blockade detection and transduction detection for examination of DNA interactions and conformational changes; and (3) channel current cheminformatics and the Utility of Nanopore Transduction for Drug Discovery.

### Transcription factor drugs

TFs are proteins that regulate gene expression by binding to the promoter elements upstream of genes to either facilitate or inhibit transcription [10,11]. They are composed of two essential functional regions: a DNA-binding domain and an activator domain. The DNA-binding domain consists of amino acids that recognize specific DNA bases near the start of transcription. TFs are typically classified according to the structure of their DNA-binding domain, which are of one of the following types: zinc fingers, helix-turn-helix, leucine zipper, helix-loop-helix, and high mobility groups. The activator domains of TFs interact with the components of the transcriptional apparatus and with other regulatory proteins, thereby affecting the efficiency of DNA binding. A cluster of TFs, for example, is used in the preinitiation complex (PIC) that recruits and activates RNA polymerase. Conversely, repressor TFs inhibit transcription by blocking the attachment of activator proteins.

Therapeutic drugs based on STFs represent a compelling new approach to the regulation of Cancer, AIDS, and genetic diseases. The creation of STFs that can appropriately target their transcription factor binding sites on native genomic DNA provides a means to directly influence cellular mRNA production (e.g. to induce death or dormancy for cancer and AIDs cells, or restore proper cellular function in the case of genetic disease). Since the cognate TF for many binding sites remains unidentified, an automated method for screening among candidates would be a highly valuable contribution to the manufacture of medicinal TFs. Developed, as it is, to study single molecule interactions/blockades on a nanometer-scale, the nanopore detector is an ideal choice for such a task. Nanopore-based research of transcription factor binding could afford the means to quantitatively understand much of the Transcriptome. This same information, coupled with supplementary interaction information upon introduction of STFs, provides a very powerful, directed approach to drug discovery.

### Nanopore blockade detector

There are important distinctions in how a nanopore detector can function: direct vs. indirect measurement of static, stationary, dynamic (possibly modulated), or nonstationary channel blockades. A nanopore-based detector can *directly *measure molecular characteristics in terms of the blockade properties of individual molecules – this is possible due to the kinetic information that is embedded in the blockade measurements, where the adsorption-desorption history of the molecule to the surrounding channel, and the configurational changes in the molecule itself directly, imprint on the ionic flow through the channel [5,6,9,12-14]. This approach offers prospects for DNA sequencing and single nucleotide polymorphism (SNP) analysis [5]. So far this is a very brief and limited synopsis of the Nanopore Detector background relevant to this paper. For other references on Nanopore Detectors see the Nanopore Detector review presented in [15]: early work involving alpha-Hemolysin Nanopore Detectors can be found in [1-6,12-14,16-23]; rapidly growing research endeavors on Nanopore Detectors based on solid-state, and other synthetic, platforms can be found in [24-34].

### Nanopore transduction detector

The nanopore-based detector works *indirectly *if it uses a reporter molecule that binds to certain molecules, with subsequent distinctive blockade by the bound-molecule complex. One example of this, with the established DNA experimental protocols, is the exploration described here of transcription factor binding sites via the different dsDNA blockade signals that occur with and without DNA binding by a hypothesized transcription factor. The modulatory auxiliary molecule represents a new "wrench in the works," a wrench that happens to rattle around in a useful fashion to create a new, much more sensitive, overall mechanism – one where it is possible to ***transduce ***single molecule events (such as intermolecular binding, intramolecular conformational change, and conformationally-mediated binding) into changes in the ionic current *stationary statistics*. More than an on/off event detection, the transduction is analog, with a range of values, such as binding strength available from binding event dwell time distributions (where event dwell times correspond exactly with the dwell time in an associated phase of ionic current stationary statistics). (Note: In the case of a direct blockage, the dwell-state could simply be a fixed blockade level, albeit noisy, with dwell time simply the time until the blockade level changes.

### Cheminformatics

The use of FSAs and Spike Detector, and HMM-EM in [5], provides a robust method for analysis of channel current data. The spike detector software [35] is designed to count "anomalous" spikes, i.e., spike noise not attributable to the gaussian fluctuations about the mean of the dominant blockade-level. The signal processing architecture is designed to rapidly extract useful information from noisy blockade signals using feature extraction protocols, wavelet analysis, Hidden Markov Models (HMMs) and Support Vector Machines (SVMs). For blockade signal acquisition and simple, time-domain, feature-extraction, a Finite State Automaton (FSA) approach is used [36] that is based on tuning a variety of threshold parameters (the spike feature extraction is obtained at this point). A generic HMM can be used to characterize current blockades by identifying a sequence of sub-blockades as a sequence of state emissions [5,11,37]. The parameters of the generic-HMM can then be estimated using a method called Expectation/Maximization, or 'EM" [38], to effect de-noising. The HMM method with EM, denoted HMM/EM, is used in what follows (further Background on these methods can be found in [5,11,37,38]). The feature vector has either 150 components or 151 if spike frequency feature included. (Further details on the 150 HMM components is given at the end of this section.) Classification of feature vectors obtained by the HMM for each individual blockade event is then done using SVMs, an approach which automatically provides a confidence measure on each classification.

The Nanopore Detector is operated such that a stream of 100 ms samplings are obtained. Each 100 ms signal acquired by the time-domain FSA consists of a sequence of 5000 sub-blockade levels (with the 20 μs analog-to-digital sampling). Signal preprocessing is then used for adaptive low-pass filtering. For the data sets examined, the preprocessing is expected to permit compression on the sample sequence from 5000 to 625 samples (later HMM processing then only required construction of a dynamic programming table with 625 columns). The signal preprocessing makes use of an off-line wavelet stationarity analysis.

### Biological significance and drug discovery utility

A new form of binding analysis/characterization is possible at the level of an individual molecular complex. This has direct relevance to biology and medicine since it can potentially provide a very direct form of binding analysis. Such a method is needed because information about molecular species, particularly their binding properties in the presence of cofactors and adjuvants, is what is often sought in drug discovery, a very complex and expensive design/discovery process. Nanopore transduction detection, thus, holds promise for helping to identify medicinal co-factors or adjuvants that lead to stronger immune response by aiding the design/selection for specific binding properties. In the results that follow TBP-TATA binding is examined (an instance of a TF/TFBS, or transcriptome, analysis), and Integrase-HIVDNA binding is examined.

## Results

### Nanopore transduction platform and transduction "carrier" signal

**Figure 1:**
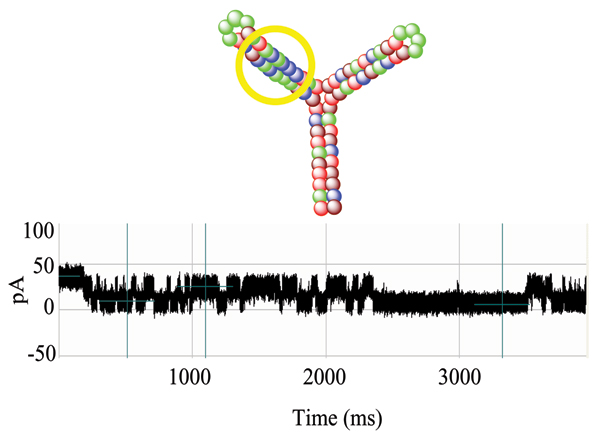
Blockade signal for Y-aptamer, T-Y10T1-GC, with TATA box (circled). A two-level dominant blockade signal is shown, preliminary discussion of this signal was given in [42].

Upon addition of the alpha-hemolysin monomers to the *cis*-well, according to the standard nanopore protocol, the toxin oligomerizes to form a water-filled transmembrane channel in the phospholipid bilayer (which blankets the limiting aperture). Next, the TY10T1-GC aptamer was applied through refluxing to this environment and began to engage the alpha-hemolysin channel. Upon capture of a single TY10T1-GC aptamer at the channel there is an immediate and overall current reduction. Thereafter, the steady flow of ions through the channel was alternately blockaded at levels corresponding to approximately 40% and 60% of baseline, hypothesized to correspond with the binding/unbinding of the aptamer's blunt-ended terminus to the surrounding vestibule walls. These fluctuations in ionic current were measured and recorded as a blockade pattern. The two-level dominant blockade signal is shown in Fig. 1 for T-Y10T1-GC.

### Aptamer-TBP model and aptamer-integrase model

In an attempt to demonstrate the nanopore detector's capacity for describing the transcription factor/transcription factor binding site interaction, we examined the TBP/TATA box complex following the nanopore protocol. TBP, a subunit of transcription factor TFIID, was selected for its broad commercial availability and nominal price. TFIID is the first protein to bind to DNA during the formation of the pre-initiation transcription complex of RNA polymerase II (RNA Pol II). The TATA box, located in the promoter region of most eukaryotic genes, assists in directing RNA Pol II to the transcription initiation site downstream on DNA. For our transduction molecular system, the TATA box is located on a 4dT-loop terminating arm of our Y-aptamer, which was prepared in the lab by annealing to two DNA hairpin molecules. The base stem of our bifunctional Y-aptamer is designed to target and bind the area around the limiting aperture of the alpha-hemolysin channel, while the arm containing the TATA box binds the TBP.

**Figure 2:**
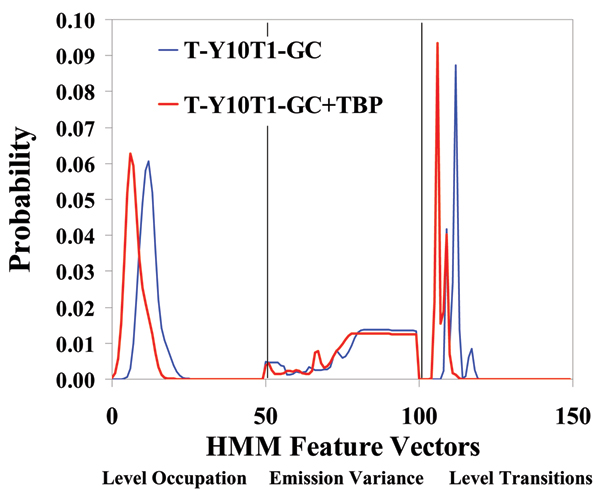
Standard 150-component HMM-based feature extraction for collections of T-Y10T1-GC blockade signals, w/wo TBP. After the EM iterations, 150 parameters are extracted from the HMM. The 150 feature vectors obtained from the 50-state HMM-EM/Viterbi implementation in [5] are: the 50 dwell percentage in the different blockade levels (from the Viterbi trace-back states), the 50 variances of the emission probability distributions associated with the different states, and the 50 merged transition probabilities from the primary and secondary blockade occupation levels (fits to two-state dominant modulatory blockade signals).

**Figure 3:**
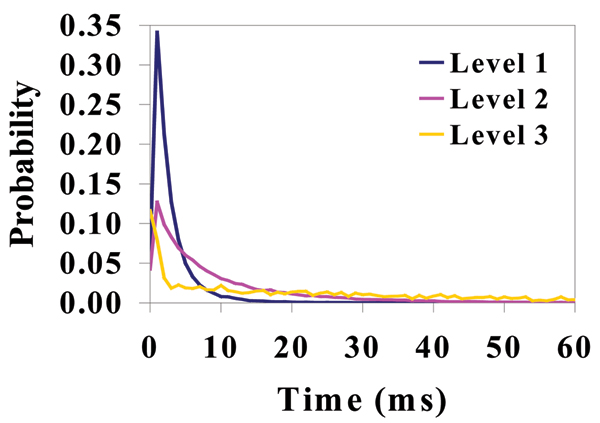
Dwell Time at Each Level for T-Y10T1-GC (see Figure 1 to visually identify the three levels – with two dominating).

**Figure 4:**
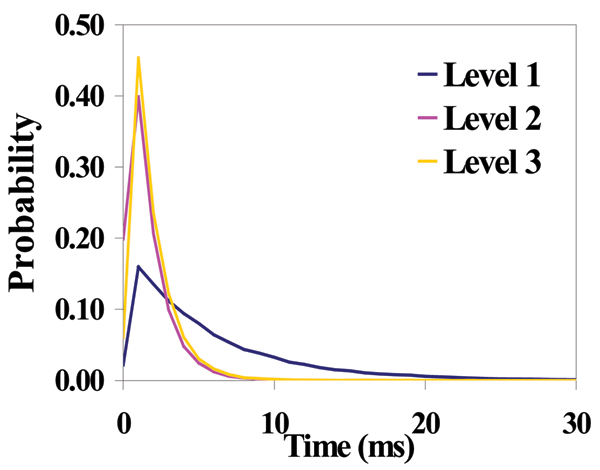
Dwell Time at Each Level for T-Y10T1-GC + TBP (sample signal blockade not shown).

We find that some of the blockade signals are only seen after introduction of TBP, which is hypothesized to be the sought after indication of TBP/TATA Box complex formation. The automated signal analysis profiles for T-Y10T1-GC w/wo TBP are shown in Figs 2, 3, 4. The experiment is also repeated (not shown), with the receptor arm elongated several base pairs for more distal receptor placement from the channel environment, in order to ensure accommodation for the TATA binding protein (TBP), with similar indication of binding in our experiments.

### DNA terminus studies: terminus dynamics

**Figure 5:**
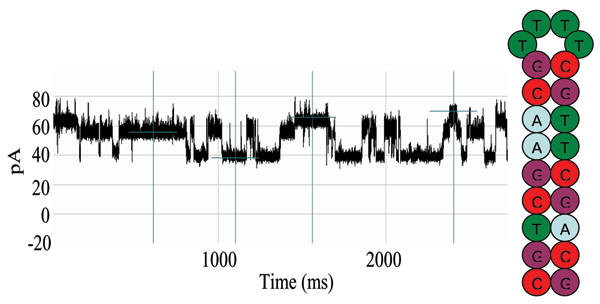
Upper Level Toggle (ULT) DNA-hairpin.

**Figure 6:**
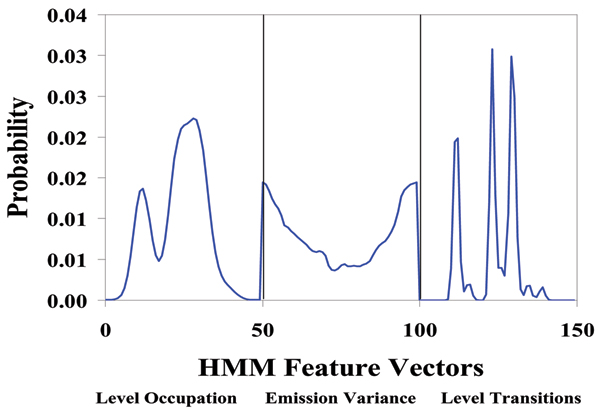
Standard 150-component HMM-based feature extraction for collections of ULT DNA hairpin (GACG-3') blockade signals. After the EM iterations, 150 parameters are extracted from the HMM. The 150 feature vectors obtained from the 50-state HMM-EM/Viterbi implementation in [5] are: the 50 dwell percentage in the different blockade levels (from the Viterbi trace-back states), the 50 variances of the emission probability distributions associated with the different states, and the 50 merged transition probabilities from the primary and secondary blockade occupation levels (fits to two-state dominant modulatory blockade signals).

**Figure 7:**
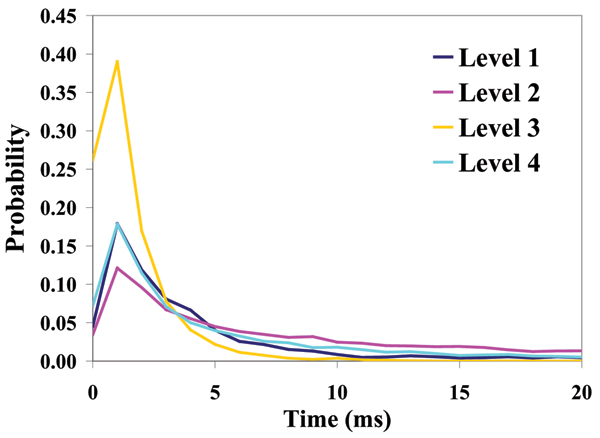
Level Dwell Time Durations for signal shown in Fig. 5.

**Figure 8:**
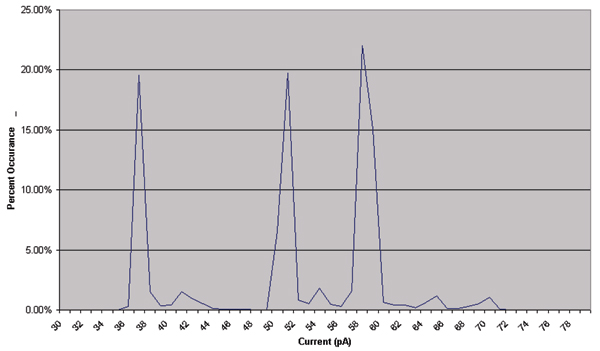
HMM Projected Level Probabilities for signal shown in Fig. 5.

DNA termini are of critical importance for certain retroviral integrases and other biological processes – being able to study them, even comparatively, offers new avenues for understanding and drug selection (HIV integrase blockers). Information on the DNA molecules' variation in structure and flexibility is important to understanding the dynamically enhanced (naturally selected) DNA complex formations that are found with strong affinities to other, specific, DNA and protein molecules. An important example of this is the HIV attack on cells. The second molecule studied focuses on the DNA terminus properties of retroviral DNA molecules. These molecules are found to exhibit greater flexibility, often marked by an increase in the number of blockade states, such as in the upper-level fine structure for the molecule terminating with GACG-3' that is examined here (see Figures 5, 6, 7, 8).

### DNA terminus studies: HIV integrase binding

One of the most critical stages in HIV's attack is the binding between viral and human DNA, which is influenced by the dynamic-coupling induced high flexibility of a CA dinucleotide positioned precisely two base-pairs from the blunt terminus of the duplex viral DNA. The CA dinucleotide presence is a universal characteristic of retroviral genomes. In previous work [9], we observe unusual nanopore blockade activity for molecules with such a CA step, indicative of unusual binding or conformational activity. In [13] a DNA hairpin with a similarly behaving GA dinucleotide step is examined in the same position. Further results along these lines are shown here, with use of the latest kinetic analysis tools. As mentioned in the Introduction, the third DNA molecule studied consists of the HIV consensus terminus at the end of the Y-aptamer arm with the TATA receptor – where it is exposed for binding to integrase. Since this molecule presents another blunt-ended dsDNA for capture, it is no surprise that such events occur. The signal analysis must separate between two classes of signal associated with these two dominant forms of capture – associated with capture of the two blunt-ended DNA regions (at the base of the Y and at the end of the integrase-binding arm). With appropriate capture of the molecule at the base of the Y, this permits direct examination of protein binding to the terminal DNA region.

**Figure 9:**
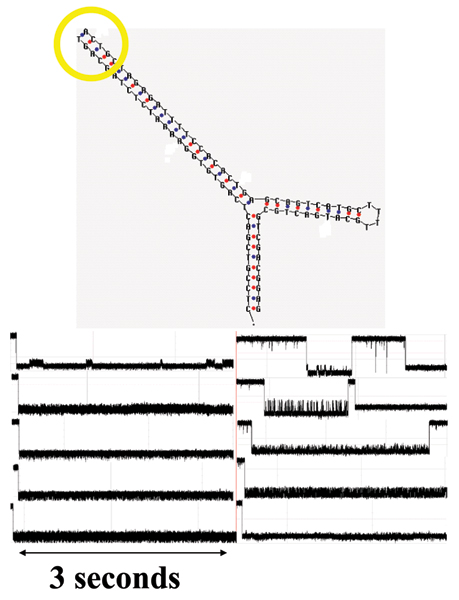
**Top**: the mfold secondary structure map of the Y-aptamer used in the integrase binding study. Integrase will bind to the blunt-ended arm shown in the yellow circle, where the HIV DNA Terminus consensus sequence has been place. **Bottom**: Blockade signals produced before (left) and after (right) introduction and possible binding of HIV Integrase to the HIV-corresponding terminus of one arm of a channel-captured y-shaped aptamer. The time elapsed during each frame is approximately three seconds.

The third molecule studied consists of a Y-aptamer with the same base sequence as in the TY10T1-GC aptamer above. The new Y-aptamer, however, has a blunt-ended arm (instead of closed with a 4dT hairpin loop) that consists of the HIV DNA terminus consensus sequence examined in the second DNA molecule experiment. The new Y-aptamer is used to observe interaction events between that terminus and HIV DNA integrase. Preliminary binding observations are shown in Figure 9. More detailed figures are shown in Additional File 1, where the three most common signal classes are shown for the HIV Y-aptamer (left side), with right side images zoomed in to a time-scale more than 100 times shorter. Similarly, a more detailed figure is shown in Additional File 2, where the a signal class is shown that is not seen when HIV Y-aptamer is introduced without addition of integrase. In that figure, a possible binding event might be shown at the change in signal pattern from fixed level that ends in the yellow box, (with actual end transition shown in the pink box).

## Discussion

### Transcription factor blocking using single-stranded oligonucleotides

The advance of drug therapies designed to target the DNA binding site of individual TFs is currently impeded by insufficient sequence specificity for the appropriate binding site. While progress is being made to produce derivatives with greater specificity, another technique for altering transcription via single-stranded oligonucleotides has been implemented in such fields of medicine as cancer treatment. This approach employs a single-stranded oligonucleotide to bind a specific sequence in double-stranded DNA and form a triple helix which is not recognizable to a protein, such as a TF, that would usually bind to that site in double helical DNA. The creation of triple helixes was used, for example, to repress the transcription of the tumor necrosis factor gene, which in turn inhibited the growth of TNF-dependent tumor cells. Despite certain successes achieved with single-stranded oligonucleotides, synthetic TFs still provide a more directed, powerful means to influence the mechanism of transcription. Nanopore technology can be play an essential role in the further development of therapeutic agents by identifying and improving structural information on the binding of these drugs to DNA. Principally, the nanopore detector gives us the ability to observe the binding event in real time with a single molecule view. Furthermore, multi-component processes not discernible by standard cell-based analysis may also be resolvable using the nanopore detector.

### Other modifications of the Y-shaped aptamer

A complementary set of experiments intended to build a foundation for a study of DNA-DNA interactions, was performed with variations of the same Y-shaped aptamer. In one experiment, the 4T loop at the end of the TATA arm was opened at the 5'-end and extended to 6T. This particular aptamer, referred to as T6-Y10T1-GC, may enter the channel at either the blunt GC terminus or the 6T overhanging terminus, thereby yielding two distinct signals representing both possible insertions. Addition of a 6A-base nucleotide sequence to the nanopore environment generated a third blockade pattern, which represented the binding event between the "sticky" 6T overhang and its complement. The blockade patterns were characterized using the nanopore detector and initial results are shown in [39].

### Further exploration of mechanism of TBP-TATA transduction signal

When TBP binds to the TATA box, it creates a nearly ninety degree bend in the DNA. This strong conformational change allows for strand separation. This is possible since the binding region of DNA is rich with the weaker two-hydrogen bond interactions of adenine and thymine. Once the strand separation occurs, RNA Pol II gains entry and begins transcription of the gene. The conformational deformity precipitated by the binding of TBP to DNA may be largely responsible for the alteration of the blockade signal originating after the introduction of TBP. Further results are sought in this area to explore the ability to observe conformational changes.

### Further exploration of retroviral DNA terminus dynamics

The behavior of the DNA hairpins containing the CA dinucleotide step (at different positions relative to their blunt-end termini), have been studied using a nanopore detector [9]. The nanopore detector feature extraction makes use of HMM-based feature extraction and SVM-based classification/clustering of "like" molecular kinetics. We have previously found that the DNA hairpin with CA dinucleotide, positioned two basepairs from the blunt terminus, has "outlier" channel current statistics qualitatively differentiable from the other DNA hairpin variants. We have also verified the same for molecules with a GA dinucleotide step (part of an earlier study, [13], as well as here). We are now attempting to explore hairpins with base changes at the same dinucleotide step (two base-pairs from the blunt ended terminus). With recent HMM methods to resolve blockade fine-structure using EVA projection, and with an HMM w/Duration implementation, we expect to fully resolve the different hairpin signal classes and will then seek to cluster them. We hypothesize that molecules with greater flexibility and added binding configurations will cluster with like molecules.

### HMM/SVM clustering for knowledge discovery

HMM-based feature extraction and SVM based clustering methods have been developed to identify clusters of duplex termini according to their blockade patterns [5,12-14]. We hypothesize that this clustering will allow us to infer useful details about the binding/conformational properties of molecules by blockade-class association. As a prelude to extensive studies of DNA annealing, Y-aptamers have been examined in a parallel study, where a ssDNA overhang (6Ts) are placed at the end of one of the Y-aptamer arms (instead of the 4dT loop cap), see [39] for further details.

### Luciferase transcription reporter vs. nanopore detector based screening

Luciferase protein can be used as an agent for measuring the ability of various therapies to stimulate or repress transcription. This is the main alternative to our proposed nanopore-based screening method. In this approach, the luciferase-encoding gene is simply linked to the gene promoter of interest, and then the entire construct is introduced into cells, where the effect of potential drug therapies (e.g., synthetic TFs) on the activity of the promoter may be discerned by comparing the expression of the luciferase protein read by luminometer in treated and untreated cells [40]. Both of the mechanisms in question have the advantage of high throughput and are capable of evaluating a broad range of compounds. However, as demonstrated in our study of the TBP/TATA complex, the nanopore device allows the binding event to be observed directly, whereas the luciferase protein would only represent its products. Moreover, the expression of luciferase does not indicate at all the manner in which a particular identified compound modulates promoter activity. Given its single molecule view, the nanopore-mechanism is capable of a superior resolution of molecular interaction.

## Conclusion

The utility and sensitivity of the Nanopore Detector when operating in "transduction mode" is described. It was hypothesized that binding targets on the transduction molecule, that are well away from the channel-captured region, could be monitored insofar as their binding status, or history, is concerned. This appears to be true for observations of binding status on the DNA molecules studied here. Extended observations, to establish the ability to monitor binding histories, is a work-in-progress.

## Methods

### Nanopore experiments

Each experiment is conducted using one α-hemolysin channel inserted into a diphytanoyl-phosphatidylcholine/hexadecane bilayer across a, typically, 20-micron-diameter horizontal Teflon aperture, as described previously [5,6]. The α-hemolysin pore has a 2.0 nm width allowing a dsDNA molecule to be captured while a ssDNA molecule translocates. The effective diameter of the bilayer ranges mainly between 5–25 μm (1 μm is the smallest examined). This value has some fluctuation depending on the condition of the aperture, which station is used (each nanopore station, there are four, has its own multiple aperture selections), and the bilayer applied on a day to day basis. Seventy microliter chambers on either side of the bilayer contain 1.0 M KCl buffered at pH 8.0 (10 mM HEPES/KOH) except in the case of buffer experiments where the salt concentration, pH, or identity may be varied. Voltage is applied across the bilayer between Ag-AgCl electrodes. DNA control probes are added to the *cis *chamber at 10–20 μM final concentration. All experiments are maintained at room temperature (23 ± 0.1°C), using a Peltier device.

### Control probe design

Since the five DNA hairpins studied in the prototype experiment have been carefully characterized [5,41], they are used in the antibody (and other) experiments as highly sensitive controls. The nine base-pair hairpin molecules examined in the prototype experiment share an eight base-pair hairpin core sequence, with addition of one of the four permutations of Watson-Crick base-pairs that may exist at the blunt end terminus, i.e., 5'-G•C-3', 5'-C•G-3', 5'-T•A-3', and 5'-A•T-3'. Denoted 9GC, 9CG, 9TA, and 9AT, respectively. The full sequence for the 9CG hairpin is 5' CTTCGAACGTTTTCGTTCGAAG 3', where the base-pairing region is underlined. The eight base-pair DNA hairpin is identical to the core nine base-pair subsequence, except the terminal base-pair is 5'-G•C-3'. The prediction that each hairpin would adopt one base-paired structure was tested and confirmed using the DNA mfold server .

### Aptamers

The DNA molecule design we are currently using consists of a three-way DNA junction created: 5'-CTCCGTCGAC GAGTTTATAGAC TTTT GTCTATAAACTC GCAGTCATGC TTTT GCATGACTGC GTCGACGGAG-3'. Two of the junctions' arms terminate in a 4T-loop and the remaining arm, of length 10 base-pairs, is usually designed to be blunt ended (sometimes shorter with an overhang). The blunt ended arm has to be carefully designed such that when it is captured by the nanopore it produces a toggling blockade. One of the arms of the Y-shaped aptamer (Y-aptamer) has a TATA sequence, and is meant to be a binding target for TBP. In general, any transcription factor binding site could be studied (or verified) in this manner. Similarly, transcription factor could be verified by such constructions, or the efficacy of a synthetic transcription factor could be examined. The other Y-aptamer, used in the integrase binding analysis, is shown in Fig. 9 (both sequence and secondary structure).

### Data acquisition

Data is acquired and processed in two ways depending on the experimental objectives: (i) using commercial software from Axon Instruments (Redwood City, CA) to acquire data, where current was typically be filtered at 50 kHz bandwidth using an analog low pass Bessel filter and recorded at 20 μs intervals using an Axopatch 200B amplifier (Axon Instruments, Foster City, CA) coupled to an Axon Digidata 1200 digitizer. Applied potential was 120 mV (*trans *side positive) unless otherwise noted. In some experiments, semi-automated analysis of transition level blockades, current, and duration were performed using Clampex (Axon Instruments, Foster City, CA). (ii) using LabViewbased experimental automation. In this case, ionic current was also acquired using an Axopatch 200B patch clamp amplifier (Axon Instruments, Foster City, CA), but it was then recorded using a NI-MIO-16E-4 National Instruments data acquisition card (National Instruments, Austin TX). In the LabView format, data was low-pass filtered by the amplifier unit at 50 kHz, and recorded at 20 μs intervals.

### Further details on the HMM cheminformatics implementation

With completion of preprocessing, an HMM is used to remove noise from the acquired signals, and to extract features from them. The HMM is, initially, implemented with fifty states, corresponding to current blockades in 1% increments ranging from 20% residual current to 69% residual current. The HMM states, numbered 0 to 49, corresponded to the 50 different current blockade levels in the sequences that are processed. The state emission parameters of the HMM are initially set so that the state j, 0 <= j <= 49 corresponding to level L = j+20, can emit all possible levels, with the probability distribution over emitted levels set to a discretized Gaussian with mean L and unit variance. All transitions between states are possible, and initially are equally likely. Each blockade signature is de-noised by 5 rounds of Expectation-Maximization (EM) training on the parameters of the HMM. After the EM iterations, 150 parameters are extracted from the HMM. The 150 feature vectors obtained from the 50-state HMM-EM/Viterbi implementation in [5,11,37] are: the 50 dwell percentage in the different blockade levels (from the Viterbi trace-back states), the 50 variances of the emission probability distributions associated with the different states, and the 50 merged transition probabilities from the primary and secondary blockade occupation levels (fits to two-state dominant modulatory blockade signals).

## Competing interests

The authors declare that they have no competing interests.

## Authors' contributions

The initial submission was written by SWH and AD, with revisions by SWH. Most of the data was gathered by AD and IA after AD was trained on nanopore detector operations by IA and EM. EM helped in constructing the nanopore device. Experimental design and pattern recognition software contributed by SWH.

## Supplementary Material

Additional file 1 The three most common signal classes are shown for the HIV Y-aptamer (left side), with right side images zoomed in to a time-scale more than 100 times shorter.
Click here for file

Additional file 2 A signal class is shown that is not seen when HIV Y-aptamer is introduced without addition of integrase. Click here for file
