# Supplementary figures and images for: Nanopore current transduction analysis of protein binding to non-terminal and terminal DNA regions: analysis of transcription factor binding, retroviral DNA terminus dynamics, and retroviral integrase-DNA binding
Source: BMC Bioinformatics. 2007 Nov 1;8(Suppl 7):S10. doi: 10.1186/1471-2105-8-S7-S10 (PMC2099478; doi:10.1186/1471-2105-8-S7-S10)

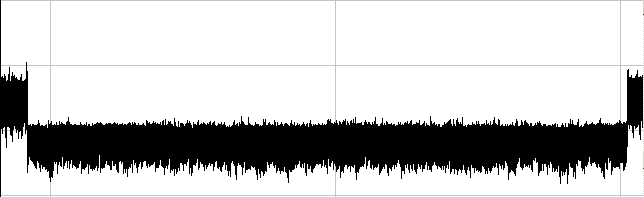

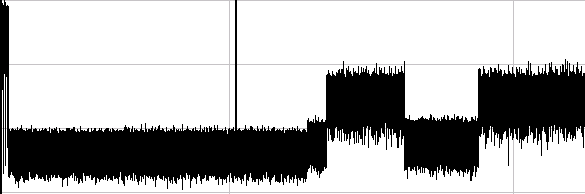

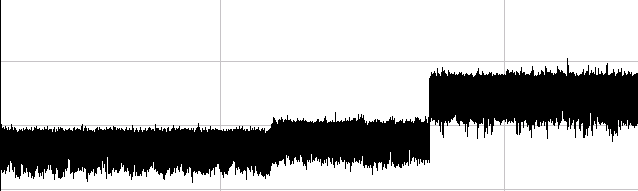


**0**

**150**

**pA**

**50 seconds**


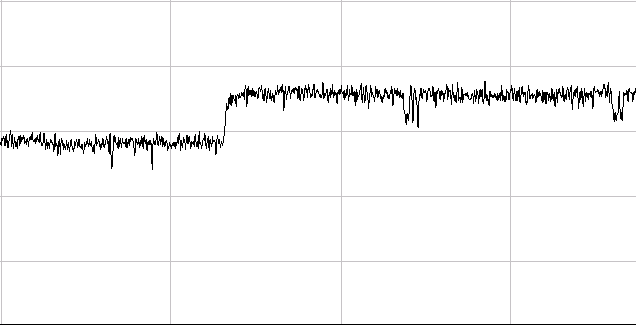

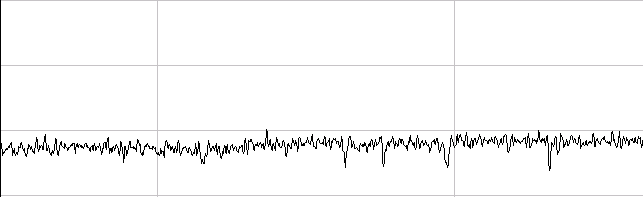


**10s**

**0.01s**

Supplement: Additional file 1 — The three most common signal classes are shown for the HIV Y-aptamer (left side), with right side images zoomed in to a time-scale more than 100 times shorter. [file 1471-2105-8-S7-S10-S1.doc]

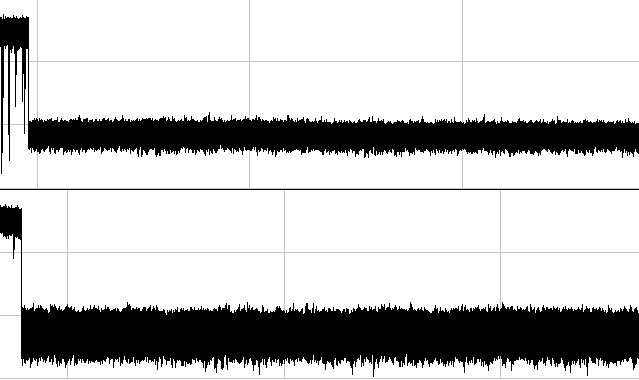

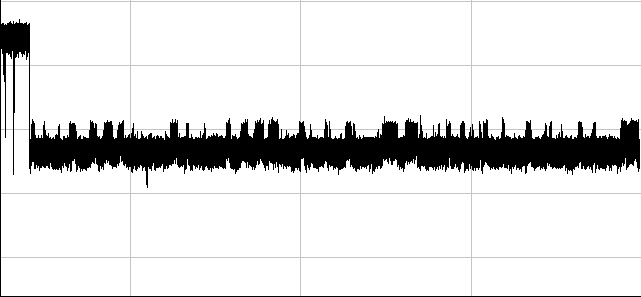

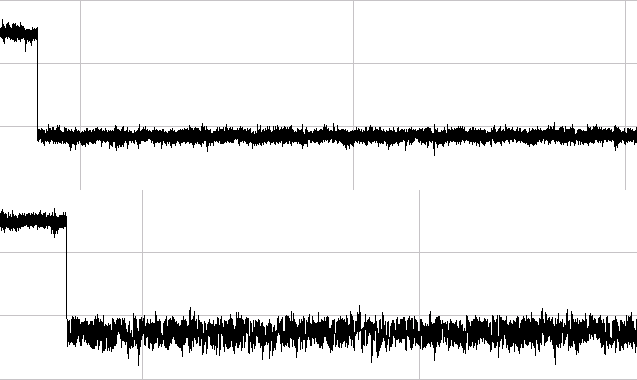

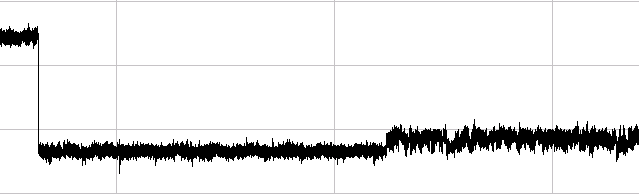


**150**

**0**

**40 seconds**

**pA**

**0.3 seconds**

Supplement: Additional file 2 — A signal class is shown that is not seen when HIV Y-aptamer is introduced without addition of integrase. [file 1471-2105-8-S7-S10-S2.doc]
